# Rebalancing mitochondrial dynamics restores succinate dehydrogenase activity and reduces succinate release in hepatocellular carcinoma

**DOI:** 10.1186/s12929-026-01289-0

**Published:** 2026-08-27

**Authors:** Jing-Yiing Wu, Tzu-Ching Chang, Mei-Jen Chen, Shih-Han Cho, Jun-Yang Liou, Cheng-Chin Kuo, Kenneth K. Wu

**Affiliations:** 1 Institute of Cellular and System Medicine, National Health Research Institutes, Zhunan, Taiwan; 2 Graduate Institute of Metabolism and Obesity Sciences, Taipei Medical University, Taipei, Taiwan; 3 Department of Bioscience Technology, Chung Yuan Christian University, Taoyuan, Taiwan; 4 Department of Medicine, National Taiwan University College of Medicine, Taipei, Taiwan; 5 Department of Pathology, Johns Hopkins University School of Medicine, Baltimore, MD USA

**Keywords:** Mitochondrial dynamics, Succinate dehydrogenase, Succinate, Dynamin-related protein 1, Hepatocellular carcinoma, Cancer metabolism

## Abstract

**Background:**

Dysregulated mitochondrial dynamics in cancer cells perturbs mitochondrial function and metabolism and promotes cancer progression. Its impacts on the electron transport chain, oxidative phosphorylation, redox balance, and glycolysis are well recognized. However, its influence on tricarboxylic acid (TCA) cycle activity is less clear. In this study, we hypothesized that excessive mitochondrial fragmentation suppresses the expression of succinate dehydrogenase (SDH), resulting in the accumulation and secretion of succinate.

**Methods:**

We tested this hypothesis in human hepatocellular carcinoma (HCC) cell model, murine xenograft tumor model, human HCC tumor tissues, and serum samples from patients with HCC. Genetic suppression and pharmacological inhibition of dynamin-related protein 1 (Drp1) were employed to examine their effects on SDH expression and succinate levels. The effects of Mdivi-1, a pharmacological inhibitor of Drp1-mediated mitochondrial fission, were evaluated in the xenograft tumor model, and the impact of succinate on mitochondrial dynamics was assessed in Huh7 cells.

**Results:**

The results reveal imbalance of mitochondrial fission and fusion proteins and increase in mitochondrial fragmentation which was associated with reduced expression of SDH and increased succinate. Succinate dehydrogenase B subunit (SDHB) mRNA levels were reduced in human HCC tumor tissues, and higher SDHB expression was associated with improved overall and relapse-free survival. Serum succinate levels were increased in patients with HCC. Genetic suppression and pharmacological inhibition of Drp1 resulted in restoration of SDH and reduction of succinate. Administration of Mdivi-1 reduced tumor growth and lung metastasis in the xenograft tumor model, which was associated with reduced p-Drp1 and increased SDHB. Addition of succinate to Huh7 cells enhanced Drp1-mediated mitochondrial fragmentation while succinate antibodies abrogated it. Overexpression of SDHB in Huh7 cells suppressed Drp1 activation and mitochondrial fragmentation through reduction of succinate. By contrast, SDHB silencing with SDHB siRNA enhanced Drp1 activation and mitochondrial fragmentation. These results suggest a positive feedback regulation of mitochondrial fragmentation by SDH/succinate.

**Conclusions:**

These findings indicate that the mitochondrial fragmentation–SDH–succinate regulatory loop plays an important role in HCC growth and metastasis and may represent a potential target for new drug development.

**Supplementary Information:**

The online version contains supplementary material available at 10.1186/s12929-026-01289-0.

## Background

Human cancer cells exhibit aberrant mitochondrial (mito) dynamics characterized by activation of dynamin-related protein 1 (Drp1) resulting in excessive mito fission and fragmentation. Increased mito fragmentation was reported to enhance cell-cycle progression and cell proliferation and to drive cancer cell migration through coordinated regulation of mito trafficking, electron transport chain (ETC), oxidative phosphorylation (OXPHOS), and aerobic glycolysis, thereby conferring advantages for cancer cell survival, proliferation, and migration [1–6]. It was reported that inhibition of mito fission reduces cancer cell growth, retards cancer migration and increases apoptosis [1–3]. In addition, dysregulated mito dynamics have been implicated in the metabolic reprogramming of cancer cells. Fragmented mitochondria exhibit altered cristae structure and respiratory activity, leading to shifts in anabolic and catabolic balance and promoting biosynthetic and redox-supportive metabolism [3, 6]. Excessive mito fission has also been associated with increased glycolytic flux, fatty acid oxidation, and disruption of mitochondrial NAD⁺/NADH homeostasis, which may collectively perturb tricarboxylic acid (TCA) cycle integrity and metabolite exchange between mitochondria and cytosol [5, 7]. Such fission-induced metabolic rewiring is thought to further promote tumor progression by sustaining energy production under metabolic stress and supporting rapid biomass accumulation. However, little is known about the influence of excessive mito fragmentation on TCA cycle intermediates and enzymes.

There is evidence for abnormal TCA cycle activities in cancer cells. For example, it was reported that cancer cells secrete succinate into the extracellular space suggesting disruption of TCA cycle. Succinate is a TCA cycle intermediate which serves as a substrate of succinate dehydrogenase (SDH) for generation of fumarate. SDH is also an integral part of ETC acting as Complex II to transport electrons from FADH2 to ubiquinone, linking the TCA cycle to ETC and OXPHOS. SDH is a protein complex comprising four subunits, i.e. succinate dehydrogenase A (SDHA), succinate dehydrogenase B (SDHB), succinate dehydrogenase C (SDHC), and succinate dehydrogenase D (SDHD). Mutations of SDHB and SDHD which are detected in hereditary and sporadic tumors notably paraganglioma and pheochromocytoma (PG/PC) lead to loss of SDH activity and accumulation of succinate [8–11]. The accumulated succinate leaks to cytoplasm where it inhibits prolyl hydroxylase and thereby stabilizes hypoxia-inducible factor 1α (HIF-1α) [10]. As HIF-1α is a master regulator of cancer growth and metastasis, succinate is considered to play a major role in PG/PC growth and metastasis. It was subsequently reported that succinate is secreted into the extracellular environment where it acts in an autocrine and paracrine manner to enhance cancer cell migration, induce angiogenesis and polarize macrophages into tumor promoting phenotypes via surface succinate receptors [12]. Succinate is now considered to be a key oncometabolite [13]. As excessive mito fragmentation and succinate accumulation are common features of human cancers, and both drive cancer growth and metastasis, we wonder whether there is a link between dysregulated mito dynamics and succinate metabolism. We tested this hypothesis in a hepatocellular carcinoma (HCC) cell model and a murine xenograft tumor model as well as human HCC tumor tissues. The results reveal that HCC cells exhibited increased mito fragmentation which was associated with reduced SDH and increased extracellular succinate. Rebalancing of mito dynamics by inhibiting Drp1-mediated mito fission restored SDH, suppressed succinate and reduced cancer growth and metastasis.

## Materials and methods

### Reagents

Succinic acid (S9515), Mdivi-1 (M0199), a pharmacological inhibitor of Drp1-mediated mitochondrial fission [14], and Carbonyl cyanide 4-(trifluoromethoxy)phenylhydrazone (FCCP; SML2959) were obtained from Sigma-Aldrich, St. Louis, MO, USA. Antibodies for SDHA (#11998, Cell Signaling Technology, Danvers, MA, USA; RRID:AB_2750900), SDHB (ab175225, Abcam, Cambridge, UK; RRID:AB_2904585), p-Drp1 (Ser616) (#3455, Cell Signaling Technology; RRID:AB_2085352), Drp1 (#8570, Cell Signaling Technology; RRID:AB_10950498), mitochondrial fission 1 (Fis1; #32525, Cell Signaling Technology; RRID:AB_2927422), mitofusin 1 (Mfn1; 13798-1-AP, Proteintech, Rosemont, IL, USA; RRID:AB_2266318), mitofusin 2 (Mfn2; #11925, Cell Signaling Technology; RRID:AB_2750893), optic atrophy 1 (OPA1; 27733-1-AP, Proteintech; RRID:AB_2810292), translocase of the outer membrane 20 (TOM20; #42406, Cell Signaling Technology; RRID:AB_2687663), FLAG (F1804, Sigma-Aldrich; RRID:AB_262044), and β-actin (MAB1501, Millipore, Billerica, MA, USA; RRID:AB_2223041) were used in Western blot analysis. Antibodies against TOM20 (11802-1-AP, Proteintech; RRID:AB_2207530) and Dylight 488-conjugated goat anti-rabbit IgG secondary antibody (#35552, Thermo Fisher Scientific, Carlsbad, CA, USA; RRID:AB_844398) were used for immunofluorescence staining.

### Patient cohort and sample collection

Tumor specimens and matched serum samples were obtained from 150 patients with HCC enrolled in a prospective, multi-center longitudinal study initiated in 2005 as part of the Taiwan Liver Cancer Network (TLCN). In addition, serum samples from 47 healthy volunteers enrolled in a previously established healthy volunteer cohort were used as controls for serum succinate analysis. All patients provided written informed consent, and the study was approved by the Institutional Review Board of National Health Research Institutes (NHRI) and Tri-Service General Hospital (IRB numbers EC1070109-E, 1-104-05-133 and 2-104-05-117). Inclusion criteria and detailed cohort characteristics have been described previously [15, 16]. Clinical data, including demographic information, tumor stage, and follow-up outcomes, were obtained from medical records and entered into a secure, de-identified database. Sample processing and storage followed standardized protocols as established by the Biobank located at NHRI, Taiwan.

### Bioinformatics survival analysis using public RNA-seq datasets

To evaluate the prognostic significance of candidate gene expression in HCC, we performed Kaplan–Meier survival analysis using the publicly available online tool Kaplan–Meier plotter (https://kmplot.com/analysis/) [17], which integrates RNA-seq–based expression profiles and clinical outcome data from The Cancer Genome Atlas (TCGA). Specifically, we selected the liver cancer RNA-seq cohort available in the Kaplan–Meier Plotter database, which comprises patients from TCGA, and analyzed overall survival and relapse-free survival endpoints using gene-specific mRNA expression values.

Patient cohorts were stratified into high and low expression groups based on the auto select best cutoff algorithm implemented by the Kaplan–Meier plotter platform, which determines the optimal threshold that yields the most significant split in survival outcome. Survival curves were generated and statistical differences between groups were assessed by log-rank test using the Kaplan–Meier plotter platform. The number of patients included in the overall survival and relapse-free survival analyses were n = 364 and n = 316, respectively.

### Cell culture and treatment

*THLE-2 cells*—THLE-2, a human epithelial cell line derived from the left lobe of a donor liver (RRID:CVCL_3803), was purchased from AddexBio (T0015001, San Diego, CA, USA). Cells were cultured in BEGM^™^ Bronchial Epithelial Cell Growth Medium Bullet Kit (CC-3170, Lonza, Walkersville, MD, USA), consisting of Bronchial Epithelial Cell Basal Medium (CC-3171, Lonza) supplemented with BEGM^™^ SingleQuots^™^ growth factors (CC-4175, Lonza).

*Huh7 cell*s—Huh7, a human HCC-derived cancer cell line (RRID:CVCL_0336), was obtained from the JCRB Cell Bank (JCRB0403, Osaka, Japan). Cells were cultured in Dulbecco’s Modified Eagle’s Medium (DMEM; 11965084, Thermo Fisher Scientific, Waltham, MA, USA) supplemented with 10% fetal bovine serum (FBS; A52567-01, Gibco, Grand Island, NY, USA), penicillin (100 U/mL), and streptomycin (100 μg/mL) (15140-122, Thermo Fisher Scientific). To evaluate the effects of succinate, Mdivi-1, or FCCP, Huh7 cells were treated with succinate (1 mM) for 24 h, Mdivi-1 (20 μM) for 6 h, or FCCP (0.5 μM) for 1 h, with untreated cells serving as controls.

*HepG2 cell*s—HepG2, a human liver cancer cell line (RRID:CVCL_0027), was obtained from the Bioresource Collection and Research Center (BCRC, RM60025, Hsinchu, Taiwan). Cells were cultured in Dulbecco’s Modified Eagle’s Medium supplemented with 10% fetal bovine serum, penicillin (100 U/mL), and streptomycin (100 μg/mL).

### Stable expression of SDHB in Huh7 cells

To generate Huh7 cells stably overexpressing SDHB, human SDHB gene was cloned into pcDNA3.1+/C-(K)-DYK (containing a C-terminal Flag tag) vector, resulting in the construct pcDNA3.1( +)-SDHB-DYK. The plasmid was obtained from GenScript, Piscataway, NJ, USA. Huh7 cells were transfected with either empty vector pcDNA3.1( +) or pcDNA3.1( +)-SDHB-DYK using X-tremeGENE™ HP DNA Transfection Reagent (XTGHP-RO, Roche Diagnostics, Mannheim, Germany), according to the manufacturer’s protocol. After 24 h, cells were subjected to selection with 40 μg/mL G418 (ant-gn-1, InvivoGen, San Diego, CA, USA), which eliminated most untransfected cells within one week. After an additional two weeks of selection, surviving cells were maintained in DMEM growth medium supplemented with 20 μg/mL G418 to establish stable cell lines.

### Succinate colorimetric assay

Huh7, HepG2, and THLE-2 cells were seeded at a density of 3.5 × 10^5^ cells/well in 6-well culture plates with 1 mL of culture medium. After cell attachment, cells were treated as indicated for an additional 48 h. Conditioned medium (CM) was then collected, and extracellular succinate levels were measured using a Succinate Colorimetric Assay Kit (ab204718, Abcam) according to the manufacturer’s instructions. Mouse plasma succinate concentrations were measured using the same Succinate Colorimetric Assay Kit and protocol as described in our previous study [12], in which circulating succinate levels in mouse plasma were successfully quantified.

### Succinate dehydrogenase activity assay

SDH activity was measured in Huh7, HepG2, or THLE-2 cells treated as indicated using a Succinate Dehydrogenase Activity Assay Kit (ab228560, Abcam) according to the manufacturer’s instructions.

### Mitochondrial morphology image and quantification

Cells were incubated with 200 nM MitoTracker^™^ Red CMXRos (M7512, Thermo Fisher Scientific) for 45 min at 37 °C, washed with PBS, fixed with 4% paraformaldehyde at room temperature for 15 min, and counterstained with DAPI. For experiments involving FCCP treatment, which directly disrupts mitochondrial membrane potential, mitochondrial morphology was assessed using TOM20 immunofluorescence staining rather than MitoTracker^™^ Red CMXRos to avoid potential membrane potential-dependent effects on mitochondrial labeling and segmentation. Mitochondrial morphology was imaged using a Leica TCS SP5 confocal microscope (Leica Microsystems, Wetzlar, Germany) under identical acquisition settings. Images were converted to 8-bit grayscale, background-subtracted, and spatially calibrated in Fiji (ImageJ; National Institutes of Health, Bethesda, MD, USA; https://imagej.net/software/fiji/). Single-cell regions of interest were manually selected using the freehand tool.

Mitochondrial morphology was quantified using the Mitochondrial Analyzer plugin in Fiji (ImageJ). Mitochondrial fragmentation count (MFC) was calculated as MFC = Branch end points/cell area. Tubular mitochondrial morphology parameters, including mean aspect ratio and mean branch length, were automatically quantified using the same plugin.

All mitochondrial morphology experiments were independently performed three times using separate cell culture preparations. In each independent experiment, three randomly selected microscopic fields were acquired for each condition under identical acquisition settings. A total of seven cells were randomly selected from these fields. Image analysis was performed in a blinded manner using the Mitochondrial Analyzer plugin in Fiji (ImageJ). Individual cells were used as the statistical unit for mitochondrial morphology quantification.

### Immunofluorescence staining

Cells were fixed with 4% paraformaldehyde for 15 min at room temperature, permeabilized with 0.1% Triton X-100 in PBS for 10 min, and blocked with Image-iT^™^ FX signal enhancer (I36933, Thermo Fisher Scientific) for 30 min. Cells were incubated with anti-TOM20 primary antibody for 1 h at room temperature, followed by incubation with DyLight 488-conjugated goat anti-rabbit IgG secondary antibody for 1 h at room temperature. Nuclei were counterstained with DAPI. Images were acquired using a Leica TCS SP5 confocal microscope under identical acquisition settings. Mitochondrial morphology was quantified using the Mitochondrial Analyzer plugin in Fiji (ImageJ).

### Antibody neutralization

Huh7 cells were treated for 24 h with a mouse IgG2a isotype control (02-6200, Invitrogen) or an anti-succinate monoclonal antibody which was custom-generated by GenScript as previously described [12]. A concentration of 0.5 μg/mL was selected based on the optimized neutralizing condition established in our previous study, in which the antibody was functionally validated for neutralizing extracellular succinate-mediated biological responses [12]. Cells were subsequently stained with 200 nM MitoTracker^™^ Red CMXRos, and mitochondrial morphology was analyzed as described above. Under the experimental conditions, the antibody did not exert nonspecific effects on cell viability, morphology or mitochondrial staining.

### Isolation of mitochondrial fraction

To isolate mitochondrial fractions, cells (5 × 10⁶ cells per sample) were treated under indicated conditions and then harvested. Mitochondria were isolated using a Mitochondria Isolation Kit for Cultured Cells (89874, Thermo Fisher Scientific) following the manufacturer’s instructions. Briefly, cells were lysed, and differentially centrifuged to obtain the crude mitochondrial pellet. The isolated mitochondrial fraction was immediately used for downstream analysis.

### RNA interference

Human DRP1 siRNA (4390824, Ambion, Austin, TX, USA) and a negative control siRNA (4390847, Ambion) were used. Human SDHB siRNA (sc-44088, Santa Cruz Biotechnology, Dallas, TX, USA) was used. Cells were transfected with siRNAs using Lipofectamine^™^ RNAiMAX (13778-075, Thermo Fisher Scientific) according to the manufacturer’s instructions. Lipofectamine^™^ RNAiMAX and siRNAs were separately diluted in Opti-MEM™ Reduced Serum Medium (31985062, Thermo Fisher Scientific), then combined and incubated at room temperature for 5 min to allow complex formation. The siRNA-lipid complexes were added dropwise to cells, which were incubated for 48 h before further analysis.

### Xenograft tumor model

4–6-week-old male BALB/cAnN.Cg-*Foxn1*^*nu/CrlNarl*^ mice were obtained from the National Laboratory Animal Center (NLAC, Taiwan) and housed under specific pathogen-free conditions under a 12-h light/12-h dark cycle at the Animal Center of NHRI. All mice were randomly assigned to experimental groups. Huh7 cells (5 × 10^6^ cells) were subcutaneously implanted into the dorsal thigh region of each mouse. To evaluate treatment effects, mice were intraperitoneally administered saline or Mdivi-1 (25 mg/kg) twice weekly for three weeks. Tumor volume was measured using calipers twice weekly for three weeks. Tumor volumes were calculated using the formula: V = (L × W^2^)/2 (L, length; W, width). All animal procedures were conducted in accordance with protocols approved by the Institutional Animal Care and Use Committee (IACUC) of AAALAC-accredited animal facility at NHRI (Protocol No. NHRI-IACUC-109058).

No predefined exclusion criteria were applied, and no animals were excluded from the final analyses. Outcome assessment was not performed in a blinded manner.

### Metastatic lung area quantification

Lung sections were stained with hematoxylin and eosin prior to image analysis. Quantification of metastatic regions in lung tissue sections was performed using the Trainable Weka Segmentation plugin in Fiji (ImageJ). A single trained classifier was applied to all images for consistency. For each image, the metastatic area was segmented based on pixel classification, while the total lung area was independently delineated by manual annotation. Metastatic burden was calculated as the ratio of metastatic area to total lung area. Five biological replicates (n = 5 mice per group) were analyzed, with the metastatic and total lung areas from multiple images per mouse summed prior to ratio calculation.

### Quantitative real-time PCR (qPCR)

Total RNA was extracted from Huh7 and THLE-2 cells using PureLink RNA Mini Kit (12183018A, Thermo Fisher Scientific) following the manufacturer’s protocol. cDNA was synthesized from total RNA using SuperScript III First-strand Synthesis System (18080051, Thermo Fisher Scientific). SDHA and SDHB transcripts were analyzed by Applied Biosystems ViiA^™^ 7 Real-Time PCR System (Thermo Fisher Scientific) using KAPA SYBR FAST Master Mix (2X) ROX Low (07959575001, Roche). Primer sequences used for qPCR analysis are shown below: human *SDHB*, forward, 5’–ACAGCTCCCCGTATCAAGAAA–3’; reverse, 5’–GCATGATCTTCGGAAGGTCAA–3’. The gene expression was analyzed using SYBR Green assay with *GAPDH* as internal control. Primers for human *GAPDH* are: forward, 5’– CGCTCTCTGCTCCTCCTGTT–3’; reverse, 5’–CCATGGTGTCTGAGCGATGT–3’. Target gene expression relative to GAPDH was calculated as 2^−(Ct of target gene − Ct of GAPDH)^.

### Quantification of SDHB mRNA in HCC tumor tissues

HCC cohort from the TLCN comprised 150 patients, including three etiological groups: hepatitis B virus, hepatitis C virus, and non-B/non-C. For each patient, paired cDNA samples derived from tumor tissue and adjacent non-tumorous liver tissue were analyzed (n = 150 matched pairs). *SDHB* mRNA expression was quantified by SYBR Green–based real-time PCR using β-actin (*ACTB*) as the internal control. The following primer sequences were used: human *SDHB*, forward, 5’–ACCTTCCGAAGATCATGCAGAG–3’; reverse, 5’–GTAGATTTTTGAGACCTTATTGAG–3’; Primers for *ACTB* are: forward, 5’–CAGCCATGTACGTTGCTATCCAGG–3’; reverse, 5’–AGGTCCAGACGCAGGATGGCATG–3’. Target gene expression relative to β-actin was calculated as 2^−(Ct of target gene−Ct of β−actin)^.

### Measurement of serum succinate in HCC patients

Serum succinate levels were quantified using ultra-performance liquid chromatography coupled with a G6495C triple quadrupole mass spectrometer (Agilent Technologies, Santa Clara, CA, USA). Chromatographic separation was performed on a BEH C18 column (1.7 μm, 2.1 × 100 mm; Waters Corporation, Milford, MA, USA) maintained at 50 °C. The mobile phases consisted of solvent A (water with 0.1% formic acid) and solvent B (acetonitrile with 0.1% formic acid). The linear gradient was as follows: 0–0.5 min, 1% B; 0.5–2.5 min, 1–10% B; 2.5–3.5 min, 10–35% B; 3.5–5 min, 35–100% B; 5–7.5 min, 100% B; 7.5–8 min, ramp down to 1% B; 8–11 min, 1% B. The injection volume was 2 μL. Mass spectrometry was performed in negative electrospray ionization mode using the G6495C triple quadrupole system. Data acquisition was conducted in multiple reaction monitoring mode. The precursor and product ion m/z values (117.02 and 73.1) for succinate were based on previously published methods [12]. Quantification was carried out using MassHunter software (Agilent Technologies).

### Migration assay

To assess cell migration, Huh7 cells were pretreated with or without 20 μM Mdivi-1 for 6 h. After treatment, 2 × 10^4^ Huh7 cells were seeded into the upper chambers of 24-well Transwell plates (8-μm pore size; 353,097, BD Falcon, Franklin Lakes, NJ, USA) in triplicate. The lower chamber was filled with 700 μL DMEM supplemented with 10% FBS as a chemoattractant. After 24 h migration, cells on the opposite side of the membrane were fixed using 100% methanol for 10 min and stained with 0.1% crystal violet for 15 min.

### Western blot analysis

Cell lysates were extracted from Huh7 and THLE-2 cells using RIPA buffer containing protease inhibitors and phosphatase inhibitors. The cellular proteins were electrophoretically resolved with 4–12% SDS-PAGE and transferred to polyvinylidene difluoride membrane. They were immunoblotted with desired specific antibodies.

### MTT assay

Cell viability was assessed using the MTT assay (3-(4,5-dimethylthiazol-2-yl)− 2,5-diphenyltetrazolium bromide; M2128, Sigma-Aldrich). Huh7 cells were transfected with scrambled control siRNA or SDHB siRNA for 48 h and then seeded into 96-well plates at a density of 1.5 × 10^3^ cells/well. Huh7-pcDNA or Huh7-SDHB-Flag stable cells were directly seeded into 96-well plates at the same density. Cell viability was measured at 24, 48, and 72 h. At each time point, 10 μL of MTT solution (5 mg/mL in PBS) was added to each well, followed by incubation at 37 °C for 3 h. Formazan crystals were dissolved in 100 μL of DMSO with gentle agitation for 15 min at room temperature. Absorbance was measured at 570 nm with background correction at 630 nm using a microplate reader.

### Statistical analysis

Statistical analyses were performed using GraphPad Prism version 8 (GraphPad Software, San Diego, CA, USA). Data are presented as mean ± SD or SEM as indicated in figure legends. Data distributions were assessed using the Shapiro–Wilk normality test where appropriate. Depending on the experimental design, either a paired or unpaired two-tailed Student’s t-test was used to compare two groups. One-way ANOVA followed by Tukey’s multiple-comparisons test was applied for comparisons among multiple groups. Tumor growth curves were analyzed using two-way repeated-measures ANOVA followed by Šídák’s multiple-comparisons test. Kaplan–Meier survival curves were generated to assess overall survival and relapse-free survival, and differences between groups were evaluated using the log-rank test. A p value < 0.05 was considered statistically significant.

## Results

### Reduction of tumor tissue SDHB mRNA and increase in serum succinate in patients with HCC

**Fig. 1:**
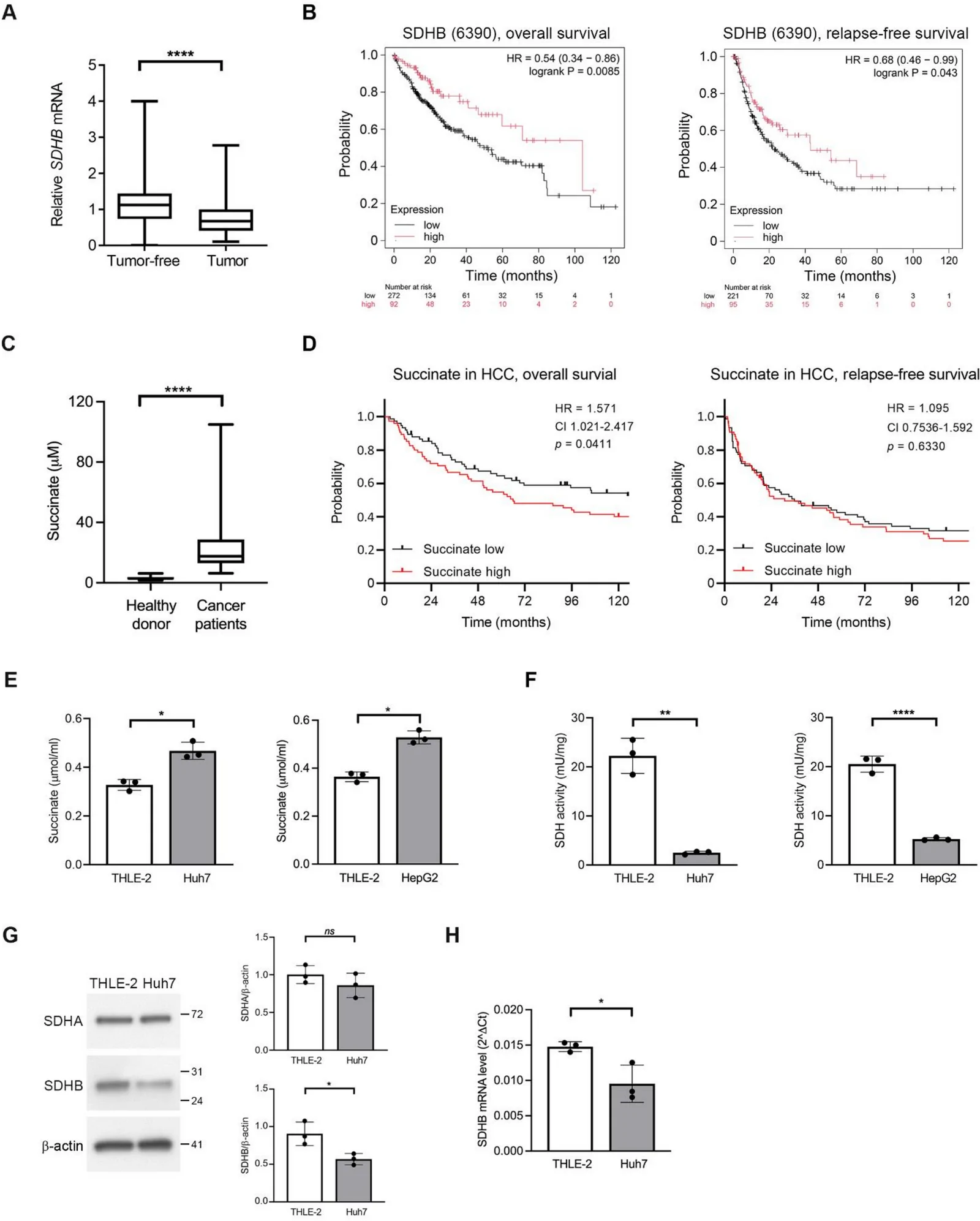
Reduced SDHB and elevated succinate levels in HCC tissues and cells. **A**–**D** Human HCC tumor tissues. **A** *SDHB* mRNA levels in paired liver tumor specimens and adjacent non-tumor liver tissues (n = 150) were quantified by qPCR. **B** Kaplan–Meier plots of overall survival (n = 364) and relapse-free survival (n = 316) stratified by low versus high SDHB expression were generated using the TCGA liver cancer dataset and the Kaplan–Meier Plotter platform with the auto-selected best cutoff. **C** Serum succinate concentrations in healthy subjects (n = 47) and HCC patients (n = 150) were measured by UPLC coupled with a G6495C triple quadrupole mass spectrometer. **D** Kaplan–Meier plots of overall survival and relapse-free survival between low- and high-succinate levels in HCC patients stratified by low versus high serum succinate concentrations (n = 150). Data in **A** and **C** represent mean ± SEM. ****P < 0.0001. Data in **B** and **D** were analyzed by log-rank test. **E**–**H** SDH and succinate in HCC cells. **E** CM was collected after 48 h of incubation. Succinate concentrations in CM from THLE-2 vs Huh7 or HepG2 cells were assessed using a Succinate Colorimetric Assay Kit. **F** SDH activity in THLE-2 vs Huh7 or HepG2 cells was measured using a Succinate Dehydrogenase Activity Assay Kit. **G** Left panel: SDHA and SDHB proteins in Huh7 vs THLE-2 cells were analyzed by immunoblotting, and right panels: densitometric quantification. **H** *SDHB* mRNA levels were quantified by qPCR. Data in **E**–**H** represent mean ± SD from at least three independent experiments. *P < 0.05; **P < 0.01; ****P < 0.0001

We analyzed tumor tissue SDHB mRNA and matched serum succinate in 150 HCC patients who enrolled in the TLCN Study. SDHB mRNA levels of tumor tissues were lower than those of the adjacent non-tumor tissues (Fig. 1A) regardless of the underlying hepatitis B and C virus status (Figure S1). Due to sample size limitation, we were unable to provide a statistically valid analysis of correlation between tumor SDHB mRNA levels and 10-year survival following therapeutic liver resection in our patient samples. However, we were able to determine the correlation by Kaplan–Meier plotter using the TCGA liver cancer database. The results show that high tumor SDHB mRNA levels were associated with significantly longer 10-year overall and relapse-free survival (Fig. 1B). Analysis of serum succinate in our patient samples (n = 150) reveal a mean serum succinate concentration of 23.3 μM (range 6.3–104.9 μM) which was higher than that of healthy subjects (Fig. 1C). The serum succinate concentration was inversely correlated with 10-year survival. Low serum succinate concentrations were associated with a longer overall survival than high succinate concentrations (Fig. 1D). However, circulating succinate levels were not significantly correlated with relapse-free survival, implying that serum succinate primarily reflects tumor aggressiveness rather than recurrence potential. These findings demonstrate that reduced tumor SDHB expression and elevated circulating succinate are both associated with poor clinical outcome in HCC. However, because tumor SDHB expression and serum succinate were not directly correlated in matched individual patients, these observations should be interpreted as complementary rather than causally linked clinical findings.

### Elevation of extracellular succinate and reduction of SDH activity and SDHB expression in HCC cells

To determine whether HCC cells release succinate into the extracellular space, we measured succinate in the CM of Huh7 and HepG2 vs THLE-2, a normal human liver cell. The succinate concentration of Huh7 CM or HepG2 CM was higher than that of THLE-2 CM (Fig. 1E). Similar to what reported in A549 cells [12], succinate enhanced Huh7 cell migration (Figure S2). As extracellular succinate accumulation could be attributed to SDH defects, we measured SDH activity of Huh7 and HepG2 vs THLE-2 cells. Huh7 and HepG2 SDH activity was significantly lower than THLE-2 SDH activity (Fig. 1F). Among the SDH subunits, SDHB deficiency was detected in several types of cancer including HCC [18]. Moreover, SDHB silencing has a profound effect on cancer cell metabolism and behavior [19]. We therefore analyzed SDHB as well as SDHA proteins by Western blotting. SDHB protein expression was reduced in Huh7 cells (Fig. 1G). SDHB mRNA level in Huh7 cells was correspondingly reduced (Fig. 1H). Together with the results from HCC patient tumor SDHB and serum succinate analysis, these findings provide evidence for reduction of SDHB expression and SDH activity and increase in succinate secretion in HCC.

### Shift of mito dynamics to mito fragmentation in Huh7 cells

**Fig. 2:**
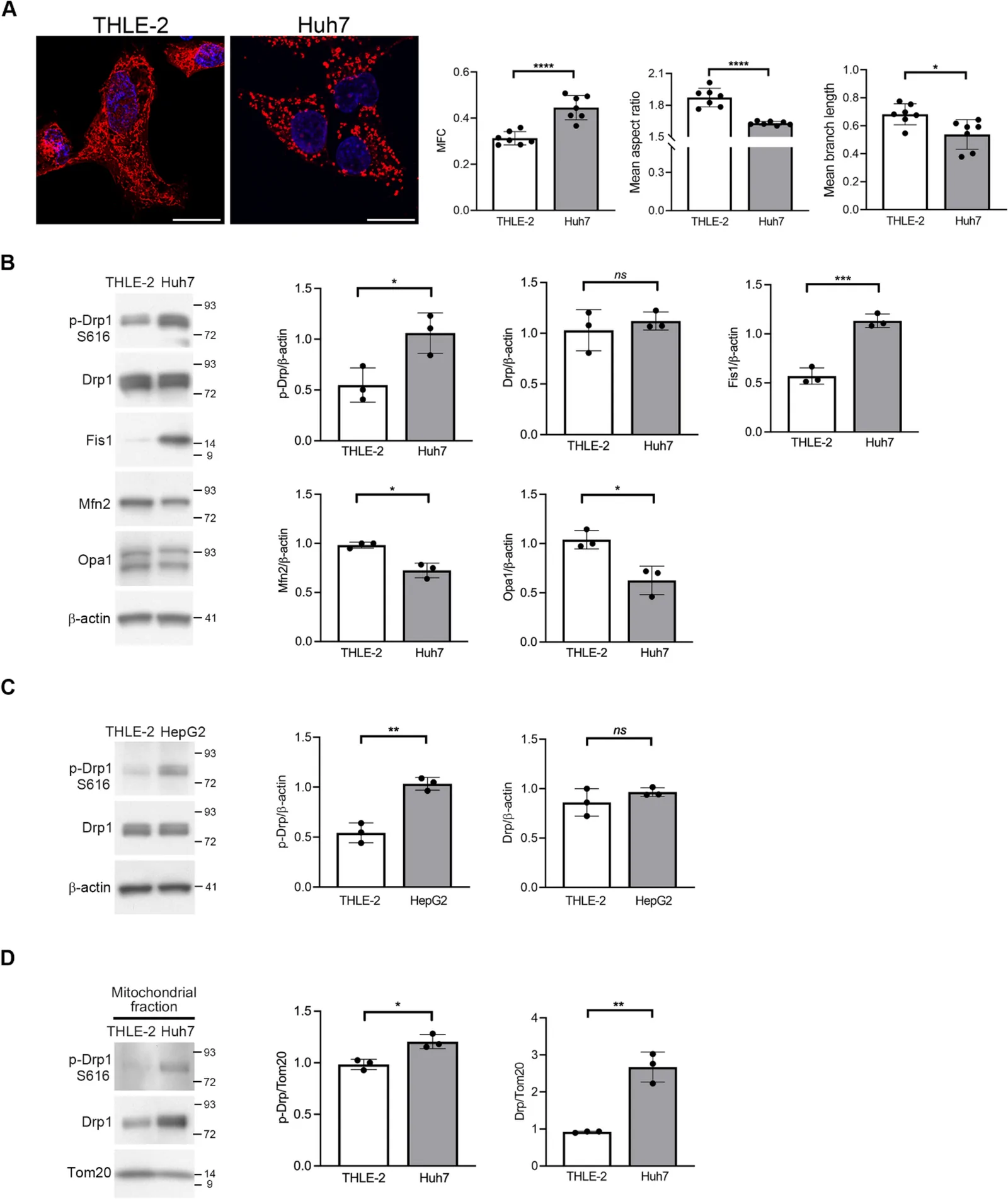
Drp1 activation shifts mito dynamics toward fragmentation in HCC cells. **A** THLE-2 and Huh7 cells were stained with MitoTracker Red CMXRos. In each experiment, images were acquired from randomly selected microscopic fields, and mitochondrial morphology was quantified in a blinded manner. A total of seven cells were randomly selected from these fields for analysis. Three independent experiments were performed. Individual cells were used as the statistical unit. Representative images are shown in the left panel and quantification of MFC, mean aspect ratio and mean branch length are shown in the right panels. Scale bar, 25 μm. **B**, **C** Total cell lysates from **B** THLE-2 vs Huh7 cells or **C** THLE-2 vs HepG2 cells were immunoblotted with antibodies against p-Drp1 (Ser616), Drp1, Fis1, Mfn2, Opa1, and β-actin in **B**, and p-Drp1 (Ser616), Drp1, and β-actin in **C**. Densitometric quantifications are shown in the right panels. **D** Mito fractions isolated from THLE-2 and Huh7 cells were immunoblotted with antibodies against p-Drp1 (Ser616), Drp1, and Tom20. Densitometric quantifications are shown on the right. Data for MFC, mean aspect ratio and mean branch length in **A** denote mean ± SD (n = 3). Data in **B**–**D** denote mean ± SD from three independent experiments. *P < 0.05; **P < 0.01; ***P < 0.001

Excessive mito fragmentation due to imbalanced mito dynamics is commonly detected in diverse cancers including HCC. We confirmed that Huh7 cells exhibited increased mito fragmentation count (MFC) and reduced tubular forms when compared to THLE-2 cells (Fig. 2A). Analysis of pro-fission and fusion factors revealed an increased level of Fis1 and reduced Mfn2 and Opa1 levels in Huh7 vs THLE-2 cells (Fig. 2B). Of note, p-Drp1 at serine616 which signifies Drp1 activation was highly elevated while Drp1 proteins were not increased in Huh7 and HepG2 vs THLE-2 cells (Fig. 2B and C). p-Drp1 and Drp1 levels were increased in the mito fraction of Huh7 cells vs normal cells (Fig. 2D) supporting translocation of activated Drp1 into the mitochondria. These results suggest that Drp1 is intrinsically activated in Huh7 cells which drives mito fragmentation.

### Extracellular succinate augmented mito fragmentation

**Fig. 3:**
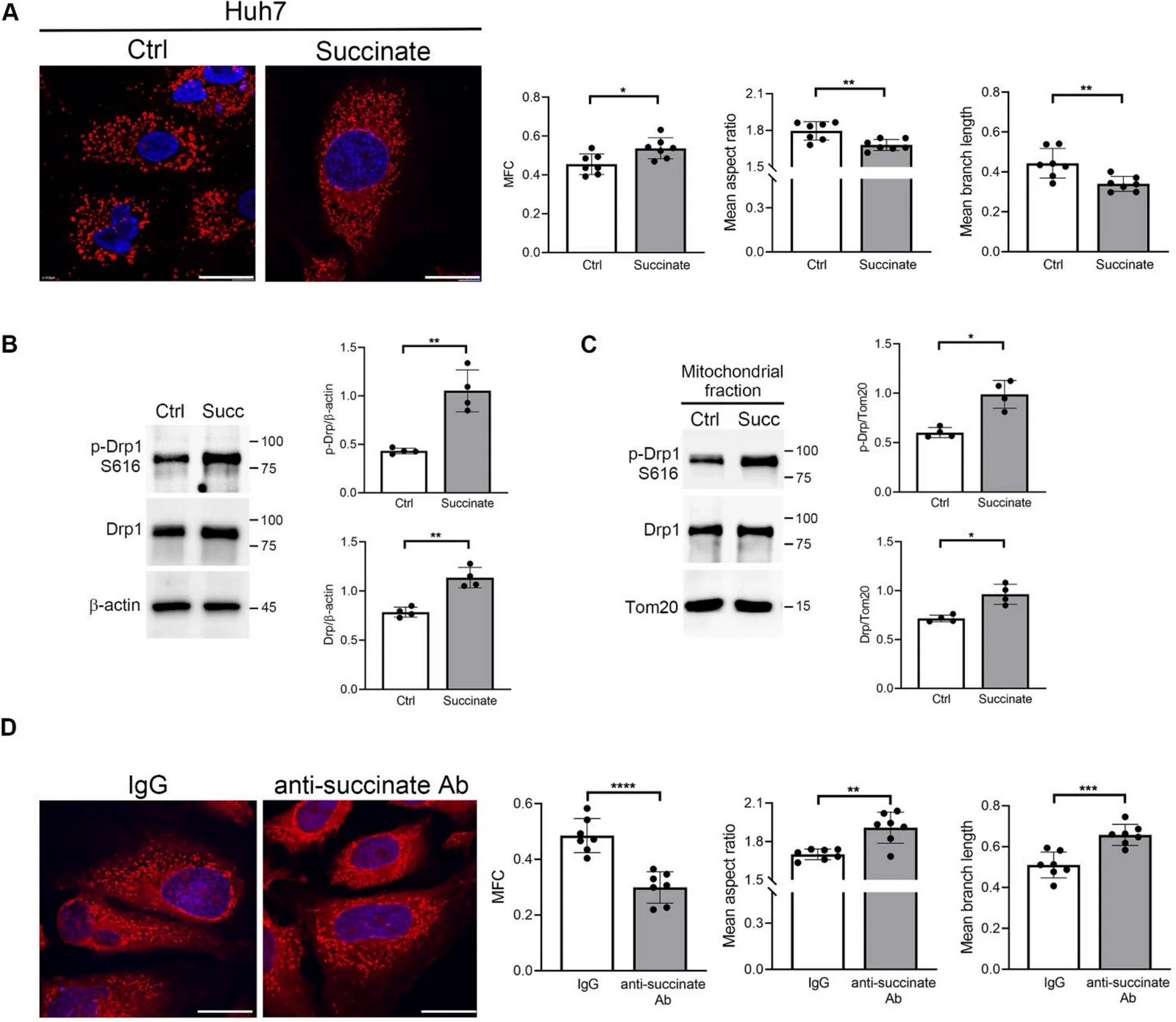
Extracellular succinate up-regulates Drp1-mediated mito fragmentation. **A**–**C** Huh7 cells were treated with succinate (1 mM) for 24 h. **A** Cells were stained with MitoTracker Red CMXRos. Representative images are shown in the left panel, and quantification of MFC, mean aspect ratio and mean branch length are shown in the right panel. Scale bar, 25 μm. **B** Total cell lysates were immunoblotted with antibodies against p-Drp1 (Ser616), Drp1, or β-actin. Densitometric quantifications are shown on the right. **C** Mito fractions from Huh7 cells were immunoblotted with antibodies for p-Drp1 (Ser616), Drp1, or Tom20. Densitometric quantifications are shown on the right. **D** Huh7 cells were treated with anti-succinate antibodies (0.5 μg/mL) or control IgG, followed by staining with MitoTracker Red CMXRos. In each experiment, images were acquired from randomly selected microscopic fields, and mitochondrial morphology was quantified in a blinded manner. A total of seven cells were randomly selected from these fields for analysis. Three independent experiments were performed. Individual cells were used as the statistical unit. Representative images are shown in the left panel and quantification of MFC, mean aspect ratio and mean branch length are shown in the right panel. Scale bar, 25 μm. Data for MFC, mean aspect ratio and mean branch length in **A** and **D** denote mean ± SD (n = 3). Data in **B** and **C** denote mean ± SD from at least three independent experiments. *P < 0.05; **P < 0.01; ***P < 0.001; ****P < 0.0001

It was suggested that succinate in the extracellular space augments mito fragmentation in cardiomyocytes under ischemia–reperfusion injury [20]. However, it is unclear whether extracellular succinate enhances Drp1-mediated mito fragmentation in cancer cells. We analyzed Drp1 mediated mito fragmentation in Huh7 cells treated with and without succinate. Succinate increased mito fragmentation (Fig. 3A), which was accompanied by increased Drp1 and p-Drp1 in whole cell lysates (Fig. 3B), and mitochondrial fractions (Fig. 3C). To determine whether endogenous succinate enhances mito fragmentation, we treated cultured Huh7 cells with succinate antibodies or control IgG. Succinate antibodies significantly reduced MFC and increased tubular forms (Fig. 3D). These results suggest that the secreted succinate from cancer cells acts in an autocrine or paracrine manner to augment mito fragmentation.

### Genetic manipulation of SDHB expression regulates mito fragmentation via succinate

**Fig. 4:**
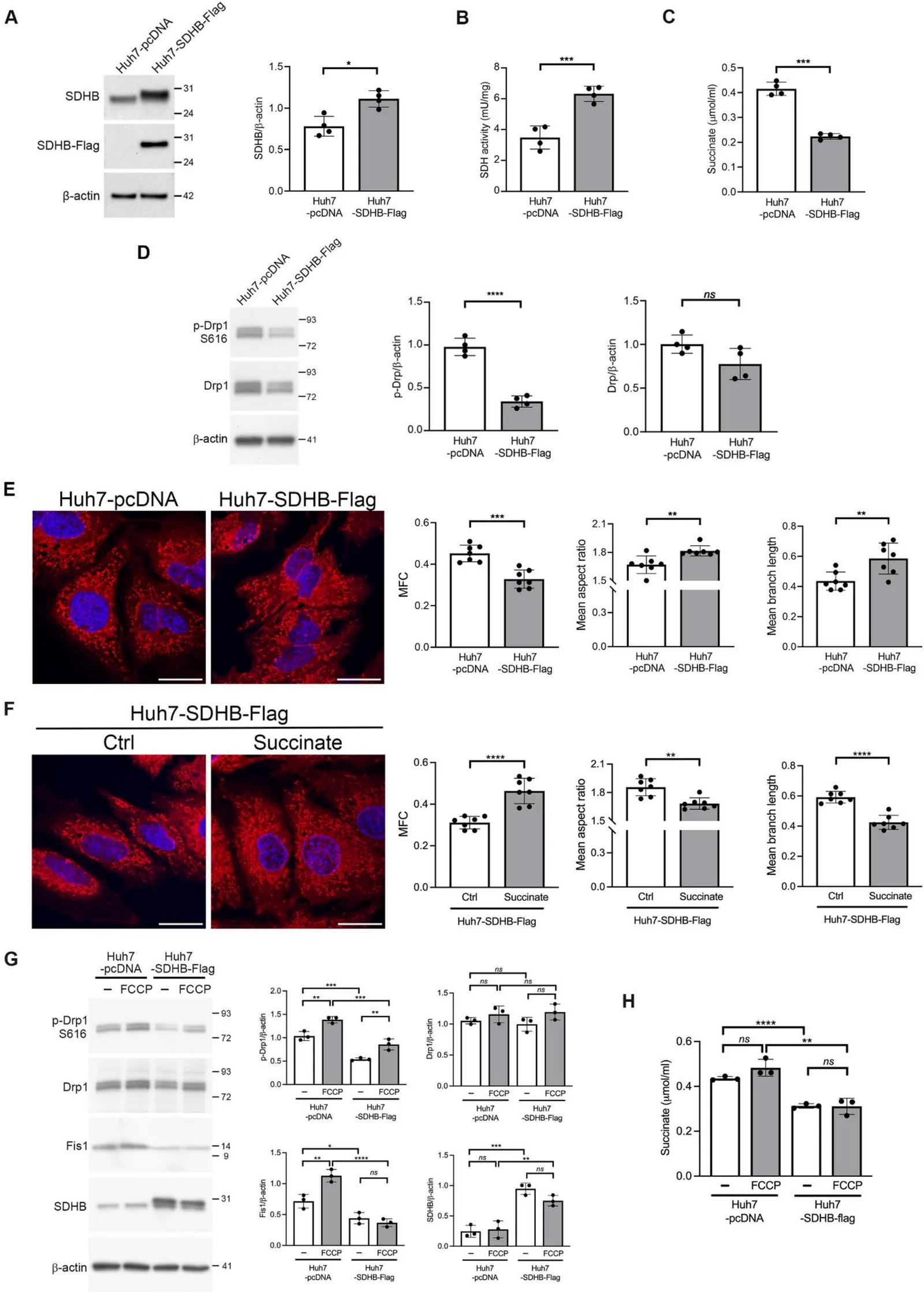
SDHB overexpression reduces CM succinate, p-Drp1 and mito fragmentation. Huh7 cells were stably transfected with control vector or SDHB-Flag. **A** SDHB protein expression was analyzed by immunoblotting. **B** SDH activity was measured using a Succinate Dehydrogenase Activity Assay Kit. **C** Following 48 h of incubation, CM was collected and succinate concentrations were measured using a Succinate Colorimetric Assay Kit. **D** Total cell lysates were immunoblotted with antibodies against p-Drp1 (Ser616), Drp1, and β-actin. Densitometric quantification is shown in the right panel. **E** Huh7 cells transfected with control vector or SDHB-Flag were stained with MitoTracker Red CMXRos. Representative images are shown in the left panel and quantification of MFC, mean aspect ratio and mean branch length are shown in the right. Scale bar, 25 μm. **F** Huh7 cells stably transfected with SDHB-Flag were treated with succinate (1 mM) or vehicle for 24 h, followed by staining with MitoTracker Red CMXRos. In each experiment, images were acquired from randomly selected microscopic fields, and mitochondrial morphology was quantified in a blinded manner. A total of seven cells were randomly selected from these fields for analysis. Three independent experiments were performed. Individual cells were used as the statistical unit. Representative images are shown in the left panel, and quantification of MFC, mean aspect ratio and mean branch length are shown in the right panel. Scale bar, 25 μm. **G** Cells were treated with FCCP (0.5 μM) or vehicle for 1 h. Total cell lysates were immunoblotted with antibodies against p-Drp1 (Ser616), Drp1, Fis1, SDHB, and β-actin. Densitometric quantification is shown in the right panel. **H** Cells were treated with FCCP (0.5 μM) or vehicle for 1 h, after which the medium was replaced with fresh culture medium. After an additional 48 h, CM was collected and succinate concentrations were measured using a Succinate Colorimetric Assay Kit. Data in **A**–**D** and **G**, **H** denote mean ± SD of at least three independent experiments. Data for MFC, mean aspect ratio and mean branch length in **E** and **F** denote mean ± SD (n = 3). *P < 0.05; **P < 0.01; ***P < 0.001; ****P < 0.0001

To provide additional evidence to support that succinate augments mito fragmentation, we transfected Huh7 cells with SDHB which resulted in enhanced SDHB expression (Fig. 4A) and SDH activity (Fig. 4B) and reduced succinate in CM (Fig. 4C), accompanied by reduction of p-Drp1 (Fig. 4D) and mito fragmentation (Fig. 4E). Of note, addition of succinate to SDHB-overexpressed Huh7 cells re-shifted the dynamic balance towards mito fragmentation (Fig. 4F). We further examined the influence of SDHB overexpression on FCCP-induced mito fragmentation. FCCP increased p-Drp1 (Fig. 4G) and mito fragmentation (Figure S3) in control Huh7 cells and its increase in Drp1-mediated mito fragmentation was suppressed by SDHB overexpression (Fig. 4G). FCCP did not significantly alter SDHB expression in SDHB-overexpressed and control Huh7 cells (Fig. 4G). It did not significantly influence CM succinate in SDHB-transfected or vector control Huh7 cells (Fig. 4H).

**Fig. 5:**
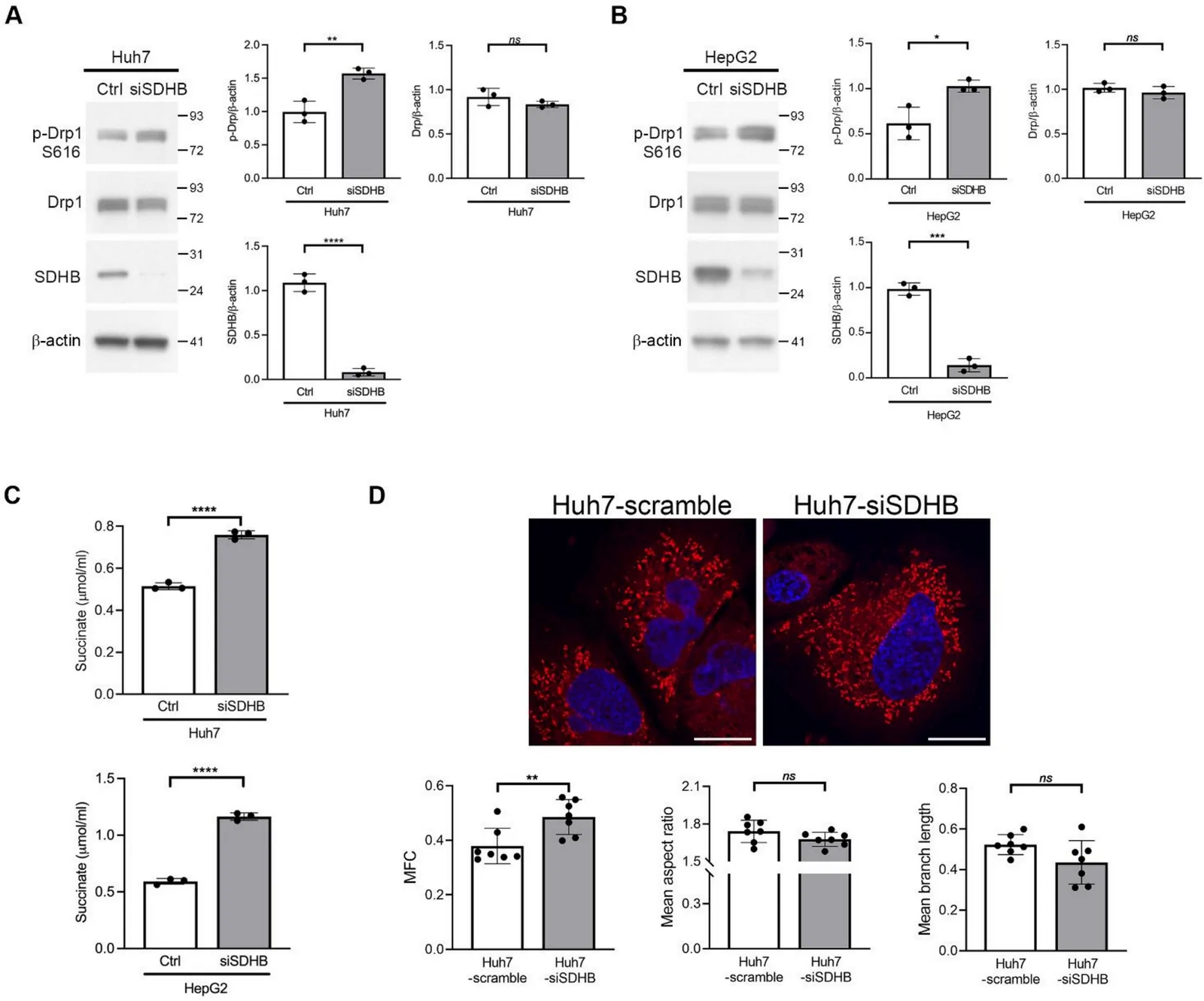
SDHB silencing by SDHB siRNA increases p-Drp1 and mito fragmentation. Huh7 or HepG2 were transfected with SDHB siRNA or scrambled control RNA for 48 h. **A** Huh7 cell lysates and **B** HepG2 cell lysates were immunoblotted with antibodies against p-Drp1 (Ser616), Drp1, SDHB, and β-actin. Densitometric quantifications are shown in the right panels. **C** Following transfection, the medium was replaced with fresh culture medium. After an additional 48 h, CM was collected and succinate concentrations were measured using a Succinate Colorimetric Assay Kit. **D** Huh7 cells were stained with MitoTracker Red CMXRos. In each experiment, images were acquired from randomly selected microscopic fields, and mitochondrial morphology was quantified in a blinded manner. A total of seven cells were randomly selected from these fields for analysis. Three independent experiments were performed. Individual cells were used as the statistical unit. Representative images are shown in the upper panel, and quantification of MFC, mean aspect ratio, and mean branch length are shown below. Scale bar, 25 μm. Data in **A**–**C** are presented as mean ± SD from at least three independent experiments. Data in **D** are presented as mean ± SD (n = 3). *P < 0.05; **P < 0.01; ***P < 0.001; ****P < 0.0001

Contrary to SDHB overexpression, SDHB silencing with SDHB siRNA resulted in an increase in p-Drp1 (S616) without a concurrent increase in Drp1 in Huh7 cells (Fig. 5A) or HepG2 cells (Fig. 5B). SDHB silencing was accompanied by increased succinate secretion into the CM in Huh7 and HepG2 cells (Fig. 5C). SDHB-silenced Huh7 cells exhibited a significant increase in mito fragmentation counts without a significant reduction of tubular forms when compared to control Huh7 cells (Fig. 5D), and increased viable cell numbers over time (Figure S4). By contrast, SDHB-overexpressed cells had reduced viable cells (Figure S4). These results support the notion that Drp1-mediated mito fission and fragmentation are regulated by SDHB expression and SDH activity and the consequent succinate accumulation and secretion.

### Restoration of SDH/succinate in Huh7 cells by Drp1 siRNA or pharmacological inhibitor

**Fig. 6:**
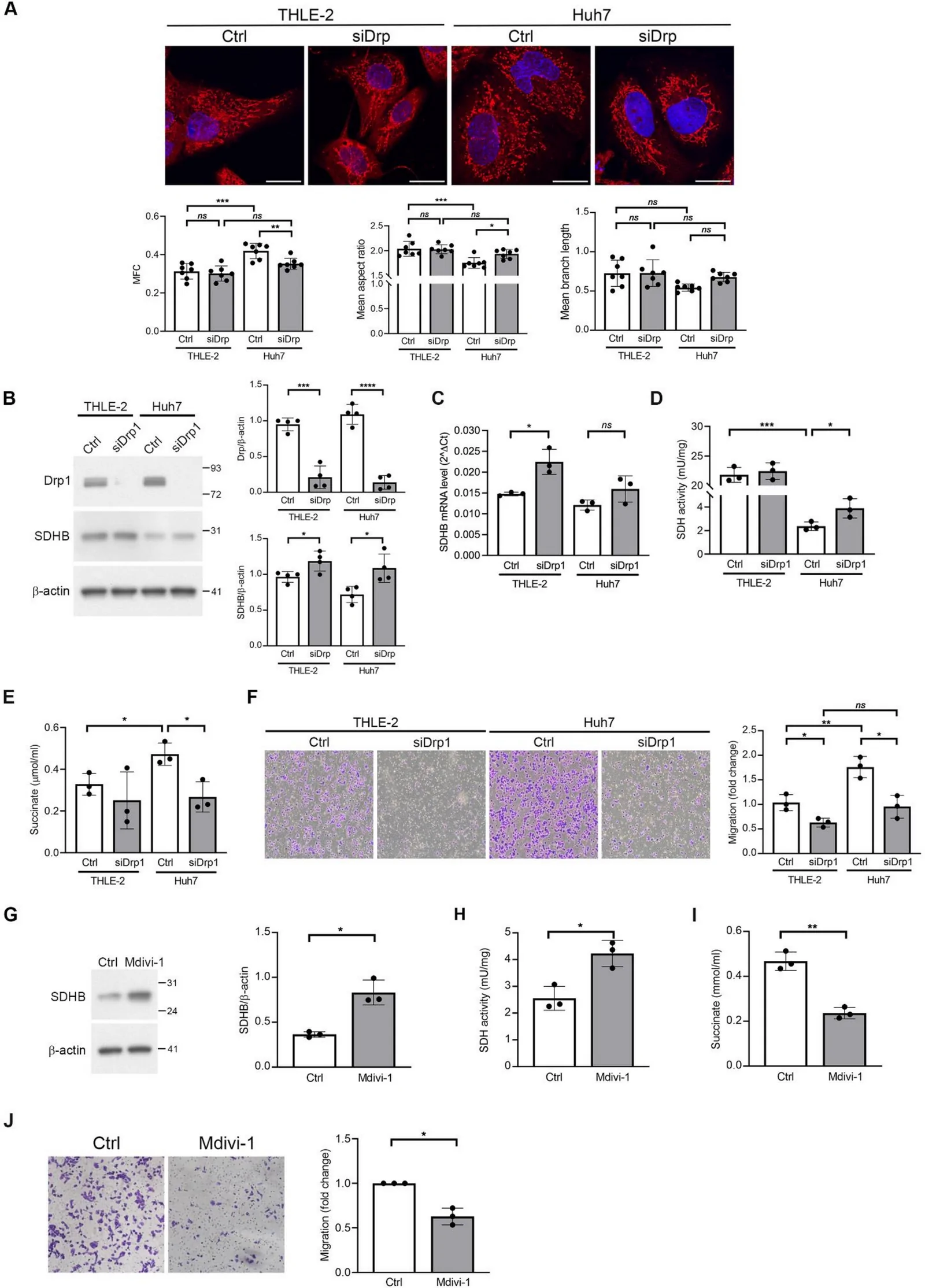
Effect of Drp1 silencing or Mdivi-1 on SDH activation, succinate release, and HCC cell migration. **A**–**F** THLE-2 and Huh7 cells were transfected with scrambled control siRNA or *DRP1* siRNA for 48 h. **A** THLE-2 and Huh7 cells were stained with MitoTracker Red CMXRos. In each experiment, images were acquired from randomly selected microscopic fields, and mitochondrial morphology was quantified in a blinded manner. A total of seven cells were randomly selected from these fields for analysis. Three independent experiments were performed. Individual cells were used as the statistical unit. Representative images are shown in the upper panel, and quantification of MFC, mean aspect ratio and mean branch length are shown below. Scale bar, 25 μm. **B** Protein levels of Drp1 or SDHB were assessed by immunoblotting. Densitometric quantifications are shown in the right panel. **C** *SDHB* mRNA levels were quantified by qPCR. **D** SDH activity was measured using a Succinate Dehydrogenase Activity Assay Kit. **E** Following transfection, the medium was replaced with fresh culture medium. After an additional 48 h, CM was collected and succinate concentrations were measured using a Succinate Colorimetric Assay Kit. **F** Cell migration was determined by Transwell assay. **G** and **H** Huh7 cells were treated with Mdivi-1 (20 μM) for 6 h. **G** SDHB protein levels were assessed by immunoblotting. **H** SDH activity was measured using a Succinate Dehydrogenase Activity Assay Kit. **I** Huh7 cells were treated with Mdivi-1 (20 μM) for 48 h. CM was collected and succinate concentrations were measured using a Succinate Colorimetric Assay Kit. **J** Huh7 cells were treated with Mdivi-1 (20 μM) for 6 h and then subjected to Transwell assays for 24 h. Cell migration was determined by Transwell assay. Data in **A** denote mean ± SD (n = 3). Data in **B**–**J** denote mean ± SD from at least three independent experiments. *P < 0.05; **P < 0.01; ***P < 0.001

To test the hypothesis that SDHB suppression in Huh7 cells is due to increased mito fragmentation, we transfected Huh7 cells with Drp1 siRNA and analyzed SDHB proteins by Western blotting and mRNAs by qPCR. Drp1 siRNA transfection reduced Drp1 protein expression in THLE−2 and Huh7 cells (Fig. 6B) which was accompanied by reduced MFC in Huh7 cells (Fig. 6A). Correlated with mito dynamic rebalancing was an increase in SDHB protein and mRNA levels (Fig. 6B and C) and corresponding to the rise of SDHB level was an elevation of SDH activity and reduction of CM succinate concentrations (Fig. 6D and E). Furthermore, Huh7 cell migration was reduced by Drp1 silencing (Fig. 6F). We next evaluated the effect of Mdivi-1, a pharmacological inhibitor of Drp1. Mdivi-1 increased SDHB protein and SDH catalytic activity (Fig. 6G and H) and reduced CM succinate (Fig. 6I). It blocked Huh7 cell migration (Fig. 6J). Together, these results provide strong evidence for disruption of TCA cycle at the SDH step by mito fragmentation resulting in generation and release of abundant succinate.

### Suppression of Huh7 cancer growth and metastasis by Mdivi-1 in a xenograft murine model

**Fig. 7:**
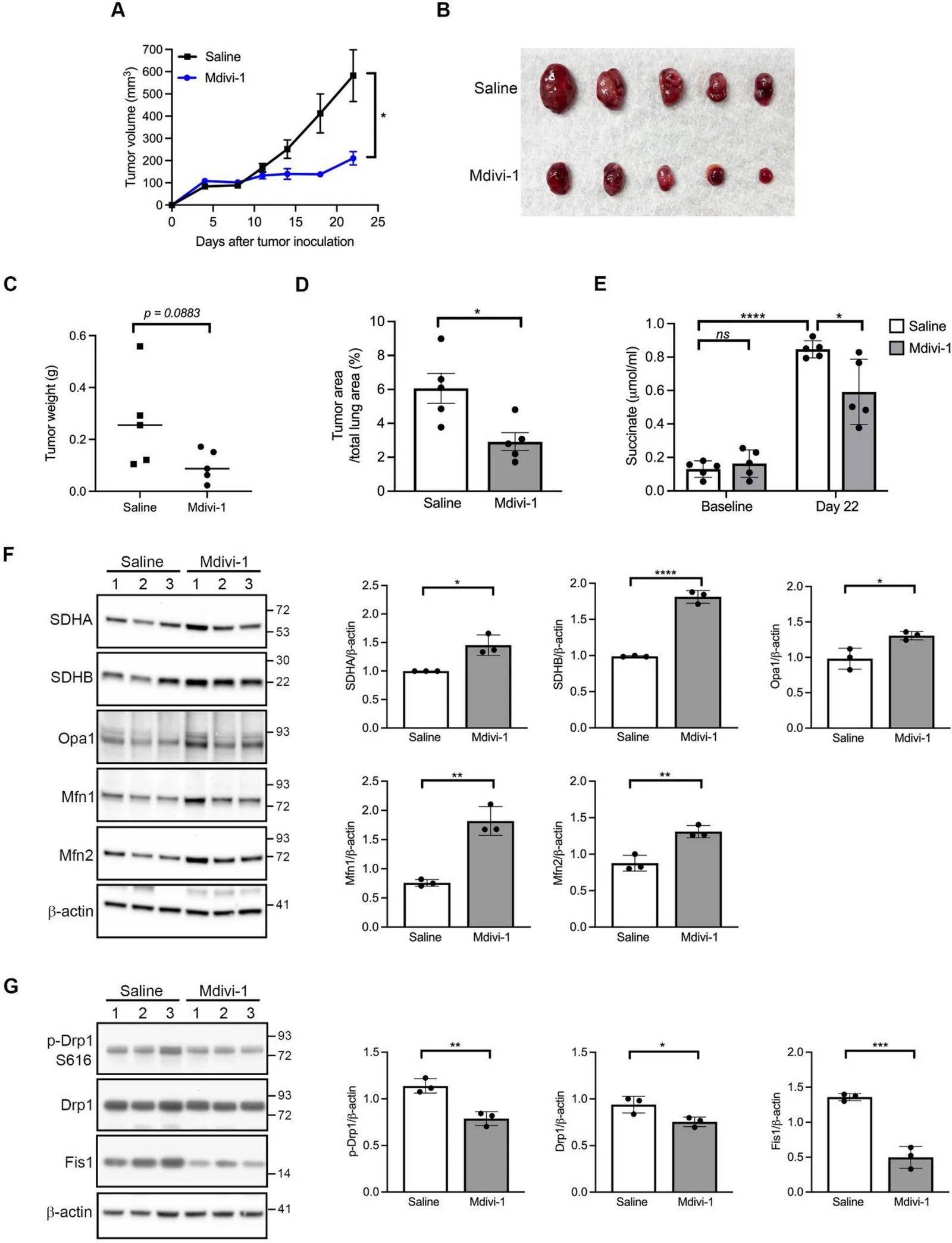
Mdivi-1 inhibits HCC tumor growth and metastasis in a xenograft murine model by restoring SDH and mito dynamics. **A**–**D** Huh7 cells were implanted subcutaneously into nude mice. Mice were treated intraperitoneally with vehicle and Mdivi-1 (25 mg/kg) twice weekly for three weeks. **A** Tumor volume was measured by calipers twice weekly. **B** Representative images of excised tumors harvested at the experimental endpoint and arranged in descending order of tumor size. **C** Tumor weight. Each dot denotes a mouse. **D** Lung metastatic area was quantified using ImageJ. **E** Plasma succinate concentration in mice before (baseline) and day 22 after Huh7 cell implantation with or without Mdivi-1 treatment. Succinate concentrations were measured using a Succinate Colorimetric Assay Kit. Data represent mean ± SEM (n = 5 for each group). **F** Protein levels of SDHA, SDHB, Opa1, Mfn1, and Mfn2 in excised tumors were assessed by immunoblotting with antibodies against the indicated proteins and β-actin. **G** Protein levels of p-Drp1 (Ser616) and Drp1 in excised tumors were assessed by immunoblotting. Densitometric quantifications for **F** and **G** are shown in the right panels. Data represent mean ± SEM of three mice per group. *P < 0.05; **P < 0.01; ***P < 0.001; ****P < 0.0001

To ensure the in vivo relevance of the cellular experiments, we evaluated the effect of Mdivi-1 on tumor growth as well as tumor expression of Drp1 and SDHB in a murine xenograft model implanted subcutaneously with Huh7 cells. Mice were treated according to the protocol illustrated in Figure S5. Intra-peritoneal administration of Mdivi-1 reduced tumor volume starting at week 2 after subcutaneous implantation of Huh7 cells (Fig. 7A). At 3 weeks, the tumor volume of the Mdivi-1 treatment group was reduced > 50% compared to the vehicle control group (Fig. 7A). Tumor size (Fig. 7B) and the resected tumor weight (Fig. 7C) at day 22 of Mdivi-1 treatment group were lower than those of the control group. We examined lung metastasis by histology. Metastatic lesions were reduced in the Mdivi-1 group (Fig. 7D). We next collected plasma samples for succinate analysis. Baseline plasma succinate levels prior to Huh7 cell implantation and 22 days after implantation with and without Mdivi-1 treatment were compared. The succinate level on day 22 was several folds higher than the baseline level, and was reduced in Mdivi-1 treated mice (Fig. 7E).

### Mdivi-1 treatment preserved tumor SDHB in the xenograft tumor model

To determine if reduction of succinate and tumor growth is related to changes in SDHB and Drp1 expression, we measured tumor SDHB, SDHA and Drp1 as well as Fis1, Mfn1, Mfn2, and Opa1 protein levels. Tumor SDHB and to a lesser extent SDHA levels were increased in the Mdivi-1 treatment group compared with the control group and Mfn1, Mfn2, and Opa1 were higher in the treatment group than the control group (Fig. 7F). By contrast, Drp1 and Fis1 levels as well as p-Drp1 were reduced in the treatment group (Fig. 7G). Data from the murine model are consistent with the results from the cellular experiments. Taken together, these findings suggest that pharmacological rebalancing mito dynamics by Mdivi-1 is associated with preservation of SDH and reduced succinate release in the xenograft model.

## Discussion

Mito fragmentation in cancer cells is associated with drastic metabolic changes including disruption of ETC, impairment of OXPHOS, increase in glycolysis and switching of fatty acid metabolism [6]. In this study, our findings suggest that mito fragmentation is associated with suppression of SDH thereby interfering with TCA cycle and inducing succinate accumulation and secretion. Our results show that inhibition of Drp1 with Drp1 siRNA or Mdivi-1 results in reduced mito fragmentation accompanied by increased SDHB protein and mRNA expression, SDH catalytic activity and reduction of succinate release into the cultured medium of HCC cells. Results from the cellular model were supported by the findings in the xenograft murine HCC model in which tumor tissue SDHB mRNA levels were increased in Mdivi-1 treated mice when compared to saline control. However, the in vivo findings should be interpreted with caution. Mdivi-1 may exert biological effects beyond Drp1 inhibition, and the reduction in plasma succinate may partly reflect the reduced tumor burden observed following Mdivi-1 treatment. Therefore, the xenograft data support the biological relevance of the proposed mitochondrial fragmentation–SDHB–succinate regulatory model but do not definitively establish a Drp1-specific causal pathway in vivo. Further, it is to be noted that inhibition of Drp1 with Mdivi-1 not only blocked Drp1 activation but also increased pro-fusion proteins including Opa1, Mfn1 and Mfn2. As Mfn2 and Opa1 may exert cellular activities independently of mito dynamics [21, 22], we cannot rule out the possibility that upregulation of SDHB may be attributed to increased Mfn2 and/or Opa1 by Drp1 inhibition. Further studies are needed to clarify the causal relationship between mito fragmentation and SDHB expression.

Our findings suggest that reduced SDHB expression in HCC cells impairs SDH catalytic activity resulting in succinate accumulation and secretion. Those findings are in keeping with the reports that SDHB mutations in PG/PC as well as SDHB silencing with siRNA in cancer cells cause loss of SDH activity and succinate accumulation [10, 11, 19]. In addition, SDHB silencing induces metabolic reprogramming and phenotypic changes [19] which contribute to cancer aggressiveness and metastasis [23]. It is unclear why SDHB expression is suppressed in cancer cells. In this study, we provide evidence that mito dynamic changes in cancer cells are associated with SDHB suppression. Our results indicate that inhibition of Drp1 with genetic silencing or a pharmacological inhibitor restores SDHB expression, increases SDH activity and reduces succinate release into the CM. As SDHB protein and mRNA levels are suppressed in HCC and both are increased by Drp1 inhibition, we reason that the defects occur at the transcriptional or post-transcriptional mRNA degradation. SDHB transcription was reported to require nuclear respiratory factor 1 (NRF1) [24]. On the other hand, accelerated mRNA degradation of SDHB could be attributable to microRNA [25]. It remains to be determined whether these transcriptional factors or microRNA are involved in SDHB suppression. How mito dynamic changes influence transcriptional or post-transcriptional regulation of SDHB expression is also unclear. It is possible that mito fragmentation and its associated mito functional perturbation such as inner membrane depolarization and mitochondrial DNA depletion may regulate nuclear gene expressions via retrograde signaling, such as increased cytosolic calcium, and MAPK activation [26, 27]. However, these potential mechanisms were not directly investigated in the present study and therefore remain speculative. Further studies are required to elucidate the underlying mechanisms.

Our results shed light on a positive feedback regulatory loop of mito fragmentation by succinate. Neutralization of extracellular succinate by succinate antibody reduces mito fragmentation while addition of succinate enhances Drp1 activation and mito fragmentation. In addition, SDHB overexpression which increases SDH activity and reduces succinate in CM increases mitochondrial tubular network while SDHB silencing with siRNA augments mito fragmentation. The positive regulatory loop amplifies and sustains mito fragmentation which enhances cancer growth and metastasis. It remains unclear how extracellular succinate activates Drp1 and promotes mito fission and fragmentation. One potential mechanism is that extracellular succinate enhances Drp1-mediated mito fragmentation via succinate receptor 1 (SUCNR1) which may transmit signals for Drp1 phosphorylation at Ser616. However, the involvement of SUCNR1 in extracellular succinate-induced Drp1 activation was not directly investigated in the present study and therefore remains to be determined.

Analysis of serum succinate in 150 HCC patients randomly selected from TLCN Study reveals a higher succinate concentration in HCC than in healthy subjects. Of note, serum succinate values in HCC patients exhibit a large variation and those with high values are at risk of shorter overall survival. However, high serum succinate values are not associated with shorter relapse-free survival. These findings suggest that HCCs with high serum succinate levels may reflect severe systemic metabolic dysregulation and are more aggressive tumor biology. However, this interpretation should be made cautiously because lack of correlation with relapse-free survival may also be due to statistical issues such as insufficient relapse events, cohort heterogeneity, or other clinical confounding factors. It was previously reported that high serum succinate in patients with PG/PC is associated with a large tumor load and a high metastatic potential [28]. Serum succinate may, thus, have the potential to serve as a prognostic biomarker for HCC and other types of cancers.

## Conclusions

In this study, we demonstrate that excessive mito fragmentation in HCC is tightly coupled to suppression of SDH, leading to pathological accumulation and secretion of succinate. In addition, we uncover positive feedback activation of Drp1 and mito fragmentation by succinate and SDH. This regulatory loop may amplify and sustain an optimal mito dynamic balance and SDH/succinate metabolism advantageous for HCC growth and metastasis. Restoration of mito dynamic balance, either genetically or pharmacologically, rescues SDH expression, reduces succinate levels, and effectively suppresses tumor growth and metastasis in vitro and in vivo. Clinical data further support the clinical relevance of this axis, as reduced tumor SDHB expression and elevated circulating succinate are each associated with poorer overall survival in patients with HCC. Collectively, these findings establish mito fragmentation–SDH dysfunction–succinate accumulation as an integrated metabolic vulnerability in HCC and highlight targeting mito dynamics and succinate metabolism as a promising therapeutic and biomarker-driven strategy for HCC intervention.

## Supplementary Information

Supplementary material 1.

## Data Availability

The datasets supporting the conclusions of this article are included within the article and its supplementary materials.

## Abbreviations

ACTB: β-Actin
CM: Conditioned medium
Drp1: Dynamin-related protein 1
ETC: Electron transport chain
FCCP: Carbonyl cyanide 4-(trifluoromethoxy)phenylhydrazone
GAPDH: Glyceraldehyde-3-phosphate dehydrogenase
HCC: Hepatocellular cancer
HIF-1α: Hypoxia-inducible factor-1 alpha
MAPK: Mitogen-activated protein kinase
MFC: Mitochondrial fragmentation count
Mito: Mitochondrial
Mdivi-1: Mitochondrial division inhibitor-1
NRF1: Nuclear respiratory factor 1
OXPHOS: Oxidative phosphorylation
PG/PC: Paraganglioma/pheochromocytoma
qPCR: Quantitative real-time PCR
SDH: Succinate dehydrogenase
SDHA: Succinate dehydrogenase A
SDHB: Succinate dehydrogenase B
SDHC: Succinate dehydrogenase C
SDHD: Succinate dehydrogenase D
TCA cycle: Tricarboxylic acid cycle
TCGA: The Cancer Genome Atlas
TLCN: Taiwan Liver Cancer Network
