# Supplementary material for: Rebalancing mitochondrial dynamics restores succinate dehydrogenase activity and reduces succinate release in hepatocellular carcinoma
Source: J Biomed Sci. 2026 Aug 27;33:86. doi: 10.1186/s12929-026-01289-0 (PMC13520282; doi:10.1186/s12929-026-01289-0)
Supplement: Supplementary file 1 [file 12929_2026_1289_MOESM1_ESM.docx]

**Supplementary data**

**Rebalancing mitochondrial dynamics restores succinate dehydrogenase activity and reduces succinate release in hepatocellular carcinoma**

Jing-Yiing Wu^1*^, Tzu-Ching Chang^1^, Mei-Jen Chen^1^, Shih-Han Cho^1^, Jun-Yang Liou^1^, Cheng-Chin Kuo^1,2,3^, and Kenneth K Wu^1,4,5*^

**Figure S1. *SDHB* mRNA levels were reduced in tumor tissues compared with adjacent non-tumor tissues, independent of hepatitis B and C virus status**

*SDHB* mRNA levels in paired liver tumor specimens and adjacent non-tumor liver tissues, stratified by HBV, HCV, or NBNC (n = 50 per group), were quantified by qPCR. HBV, hepatitis B virus; HCV, hepatitis C virus; NBNC, non-B/non-C. Data denote mean ± SEM. ***P* < 0.01; *****P* < 0.0001.

**Figure S2. Succinate enhances Huh7 cell migration**

Huh7 cells were treated with succinate (1 mM) or vehicle during a 24-h Transwell migration assay. Quantification of cell migration cells is shown in the right panel. Data denote mean ± SD from three independent experiments. **P* < 0.05.

**Figure S3. SDHB suppresses FCCP-induced mitochondrial fragmentation**

Vector control and SDHB-overexpressing Huh7 cells were treated with FCCP (0.5 μM) for 1 h and stained with anti-Tom20 antibodies. Representative images are shown in the upper panel and quantification of mitochondrial fragmentation count (MFC) and tubular mitochondrial morphology parameters, including mean aspect ratio and mean branch length, are shown below. Scale bar, 25 μm. Data denote mean ± SD from seven cells. **P* < 0.05; ***P* < 0.01.

**Figure S4. SDHB regulates Huh7 cell viability**

(A) Huh7 cells were stably transfected with SDHB. Cell viability in control Huh7 and SDHB-overexpressing Huh7 cells was measured by MTT assay. (B) Huh7 cells were transfected with scrambled control siRNA or SDHB siRNA, and cell viability was measured by MTT assay. Data denote mean ± SD from three independent experiments. **P* < 0.05; *****P* < 0.0001.

**Figure S5. Experimental design for establishing xenograft HCC models**

Huh7 cells were implanted subcutaneously into nude mice. Mice were treated intraperitoneally with vehicle or Mdivi-1 (25 mg/kg) twice weekly for three weeks. Tumor volume was measured by calipers twice weekly.
